# Comparative Systematic Review and Meta-Analysis Between Robotic and Laparoscopic Abdominoperineal Resection for Rectal Cancer: Oncological and Short-Term Outcomes

**DOI:** 10.7759/cureus.72877

**Published:** 2024-11-02

**Authors:** Huda Mohammed, Ingie M Gabra, Nouran Halawa, Saira Naeem, Cyprian O Ogah, Tuheen Sankar Nath

**Affiliations:** 1 Colorectal Surgery, Luton and Dunstable Hospital, Luton, GBR; 2 Anesthesiology, John Muir Health, Walnut Creek Medical Center, Walnut Creek, USA; 3 Internal Medicine, Ain Shams University, Cairo, EGY; 4 Internal Medicine, Faisalabad Medical University, Faisalabad, PAK; 5 Internal Medicine, Valley Baptist Medical Center, Harlingen, USA; 6 Surgical Oncology, California Institute of Behavioral Neurosciences & Psychology, Fairfield, USA

**Keywords:** abdominoperineal resection, laparoscopic surgery, minimally invasive surgery, rectal cancer, robotic surgery

## Abstract

Minimally invasive surgery, especially robotic surgery, has become increasingly popular in colorectal surgery over the last decade. Robotic-assisted surgery has shown better outcomes than conventional laparoscopic surgery because of superior ergonomics, high-resolution three-dimensional cameras, and articulating instruments; however, issues like the long operative time and cost-effectiveness remain unresolved. This study compares the robotic and laparoscopic approach of abdominoperineal resection (APR) for low rectal cancer to evaluate the superiority of robotic surgery in short-term and oncological outcomes.

This meta-analysis used Preferred Reporting Items for Systematic Reviews and Meta-Analyses (PRISMA) guidelines and principles. We conducted a systematic search in the PubMed, Cochrane, Google Scholar, and ResearchGate databases, and seven studies were included after the standardized quality check. Inclusion criteria involved any patient at the age of 18 or above with low rectal cancer who underwent APR with the exclusion of studies that are not in English, patients who underwent open surgery, and patients with recurrent cancer.

A total number of 10,331 participants from seven eligible studies were involved in this review and meta-analysis with 1499 in the robotic group and 8,895 in the laparoscopic group. The oncological outcome showed that the positive resection margin is significantly higher in the laparoscopic group with a mean difference (MD) of 0.35, 95% CI (0.14, 0.89), and P = 0.03. The difference in the number of harvested lymph nodes between the robotic and laparoscopic APR is not statistically significant with an OR of 0, 95% CI (-0.19, 0.19), and P = 0.98. The operative time was found to be higher in the robotic group with an MD of 11.25, 95% CI (9.25, 13.04), and P = 0.0001. A higher conversion rate was reported in the laparoscopic group with OR 0.38, 95% CI (0.28, 0.51), and P = 0.00001. The length of the hospital stay was less in the robotic group with an MD of 2, 95% CI (-2.08, -1.92), and P = 0.00001. A lower rate of postoperative ileus was found in the robotic group with an odd ratio of 0.74, 95% CI (0.61,0.89), and P = 0.001, which is statistically significant. The mortality rate and thromboembolic complication also showed a significantly higher rate in the laparoscopic group with an OR of 0.15, 95% CI (0.03, 0.84), and P = 0.03 and an OR of 0.14, 95% CI (0.03, 0.69), and P = 0.02, respectively. There is no statistically significant difference between the robotic and laparoscopic groups in the surgical site infection, urinary dysfunction, cardiac, and pulmonary complications.

In conclusion, our study findings indicated that robotic APR has a better short-term outcome and negative circumferential resection margins compared to laparoscopic APR. However, more research and prospective randomized controlled trials are needed to determine the efficiency, cost effectiveness, recurrence, and survival rate in robotic APR.

## Introduction and background

Of all cancers, colorectal cancer (CRC) has the second-highest incidence rate in Europe, and of the approximately 500.000 cases of CRC that occur each year, 175,000 are found in the rectum [1]. Abdominoperineal resection (APR) is a surgical procedure that involves removing the rectum, anal canal, accompanying levators, anal sphincter complex, and perineal skin, resulting in a permanent colostomy [2]. In advanced lower rectal cancer with big tumors, sphincter involvement, or difficulty establishing an acceptable distal resection margin, APR is the recommended surgical approach [3].

This procedure carries substantial risks and complications that have an immediate impact on the patient's health and long-term consequences on their social well-being, particularly in terms of permanent stoma and sexual dysfunction [4]. APR can be performed by open, laparoscopic, or robotic approaches.

Over the last decade, the demand for minimally invasive surgery has grown rapidly, with robotic-assisted surgery at the top of this development [5]. Laparoscopy became very popular since it was introduced in colorectal surgery because of its positive outcomes, including shorter hospital stays, less postoperative pain, better cosmetic outcomes, and quicker recovery after the operation [6]. The introduction and development of robotic surgery is one of the many technological advances that will have the greatest potential benefits in surgery in the future. It has demonstrated the ability to achieve results that are even better than those obtained with the laparoscopic approach, particularly in high-risk populations including obese patients, patients undergoing long procedures, and male patients [5,7].

Using a laparoscope to operate on lower rectal tumors represents several challenges, such as manipulating long, rigid laparoscopic tools due to limited flexibility, experiencing hand and tool tremors, getting a clear view in the narrow pelvis, particularly in male, obese patients, and patients undergoing neoadjuvant therapy, in addition to [8-9]. On the other hand, robotic techniques might overcome these issues by providing a three-dimensional view, more advanced ergonomics, articulated instruments, and an assistant arm [10-11].

The purpose of our study is to evaluate the differences between the robotic and laparoscopic approaches to APR for rectal cancer and to assess the effective outcomes and advantages of robotic surgery over the laparoscopic approach.

## Review

Methods and study design

**Figure 1:**
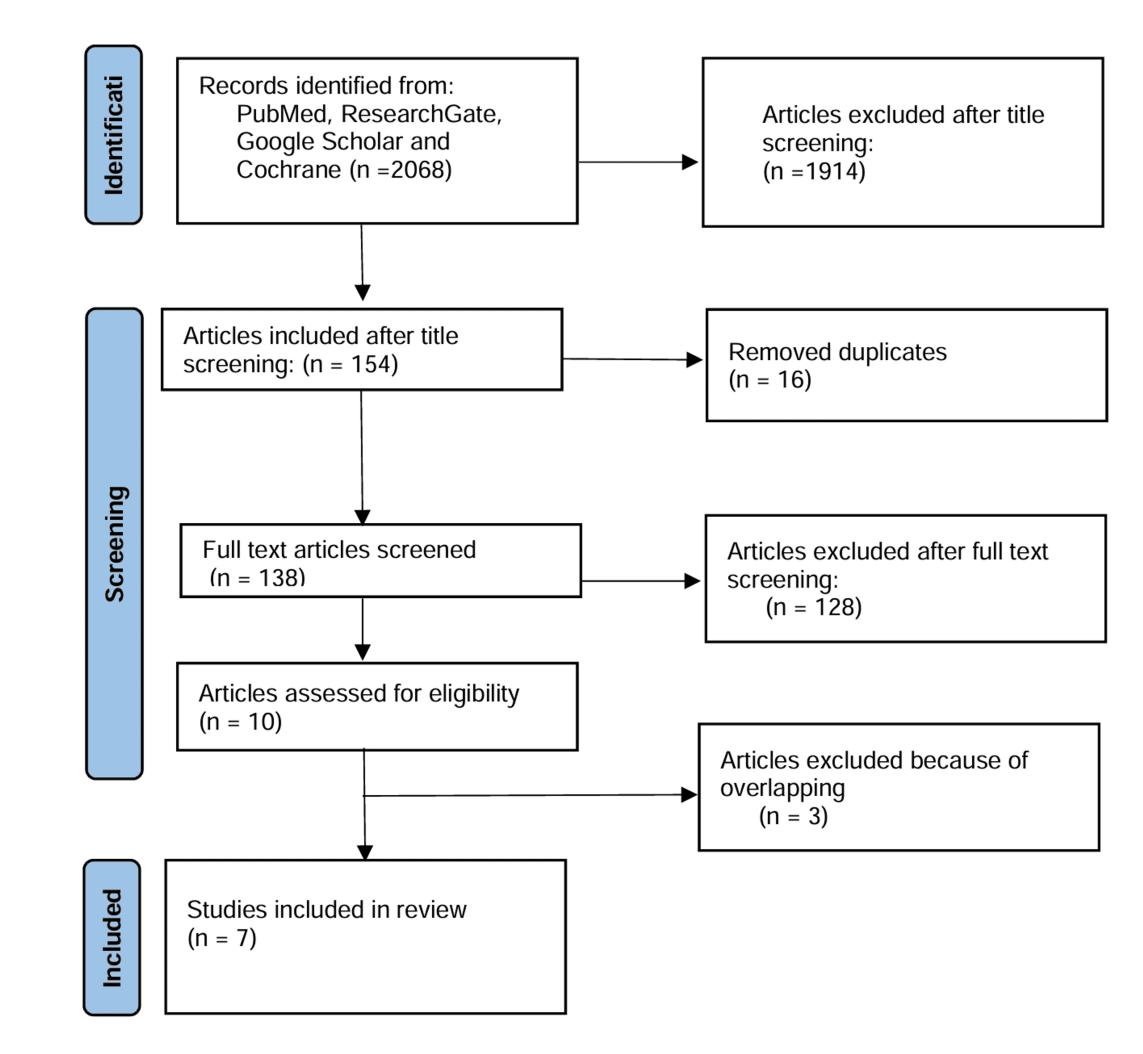
PRISMA flow chart of studies selection

This systematic review and meta-analysis were conducted by using the Preferred Reporting Items for Systematic Reviews and Meta-Analyses (PRISMA) guidelines, and principles (Figure 1) [12]. We systematically searched the PubMed, Cochrane, Google Scholar, and ResearchGate databases. Keywords, including (Robotic or robotic-assisted surgery) vs (Laparoscopic surgery or laparoscopy) (abdominoperineal resection or rectal resection) for rectal cancer, were used to identify the articles. The list of relevant articles was reviewed systematically to identify related studies. All articles were evaluated using inclusion and exclusion criteria. 

Inclusion and Exclusion Criteria

The inclusion criteria were as follows: population: all patients were the age of 18 years and above, undergoing surgery for low rectal cancer; intervention: robotic or laparoscopic abdominoperineal resection; comparison: robotic surgery versus laparoscopic surgery for abdominoperineal resection; outcome: the primary outcome of this study is the oncological outcome including circumferential resection margins, numbers of harvested lymph nodes, and recurrence rate, while the secondary outcomes include short-terms complications such as conversion rate to open surgery, surgical site infection (SSI), blood loss, operative time, hospital stay, ileus, urinary dysfunction, thromboembolic, cardiac, and pulmonary complications and mortality rate. We excluded studies not in English or those with patients who underwent open surgery, had recurrent cancer, and were aged under 18 years.

Quality Assessment of the Studies

We systematically evaluated seven studies for quality using standardized quality check tools, and all of them were included in the review as they qualified as high-quality studies. Newcastle-Ottawa scale and Cochrane risk of bias assessment tool were used for observational studies and RCT, respectively. The characteristics of the seven studies are listed in Table 1.

**Table 1:** Studies characteristics RCT: randomized controlled trial

Study	year	Type	Sample size	Robotic	Laparoscopic	Exclusion
Moghadamyeghaneh et al., [13]	2015	retrospective cohort study	5609	872	4737	age below 18, emergency surgery, Metastatic cancer
Kamali et al., [14]	2017	prospective cohort study	22	11	11	cancer other than adenocarcinoma
Gavrila et al., [15]	2021	retrospective cohort study	109	46	63	recurrence, non-experienced surgeon
Kasai et al., [16]	2021	retrospective cohort study	53	33	20	lateral lymph node dissection, simultaneous resection
Feng et al., [17]	2022	RCT	347	174	173	concomitant cancer, complete response after CRT, CT1 n0 for local excision, need emergency surgery, multiple colorectal tumors, hereditary colorectal cancer
Mizoguchi et al., [18]	2023	retrospective cohort study	4148	341	3870	unknown stage, or 0, concurrent surgery
Tatsuki et al., [19]	2024	retrospective cohort study	43	22	21	N/A

Statistical Analysis

Study data, including first author, year of publication, type of study design, patient characteristics (i.e., number of patients, age, gender, ASA score and comorbidities ), intraoperative data (i.e., operative time, blood loss, conversion to open surgery), tumor pathological data (i.e., TNM stage, lymph nodes harvested, tumor size, and resection margin positivity), and short postoperative outcomes (length of hospital stay, mortality and postoperative complications) was extracted into a Microsoft Excel sheet. Table 2 describes the patients’ characteristics.

**Table 2:** Patient's characteristics BMI: body mass index, DM: diabetes mellitus, HTN: hypertension, CRT: chemoradiotherapy

Study		Moghadamyeghaneh et al., [13]	Kamali et al., [14]	Gavrila et al., [15]	Kasai et al., [16]	Feng et al., [17]	Mizoguchi et al., [18]	Tatsuki et al., [19]
Age group (mean +SD)	Robotic	64±12	71±10.1	62.2±11	74	58.2±9.6	68.3	66.5
	Laparoscopic	62±13	57±12.7	62.25±10.9	78	59.5±10.9	70.3	67
Male	Robotic	556	7	34	20	108	212	13
	Laparoscopic	2844	9	32	16	66	2447	13
Female	Robotic	316	4	12	13	113	129	9
	Laparoscopic	1893	2	31	4	60	1360	8
BMI	Robotic	-	-	24.3±3.7	22	-	22	22
	Laparoscopic	-	-	23.5±4	23.7	-	22.2	22.1
DM	Robotic	154	-	-	9	19	49	2
	Laparoscopic	900	-	-	3	16	699	1
HTN	Robotic	409	-	-	14	49	57	-
	Laparoscopic	2232	-	-	10	66	953	-
Pulmonary disease	Robotic	87	-	-	3	2	4	-
	Laparoscopic	584	-	-	0	3	84	-
Neoadjuvant CRT	Robotic	-	7	31	5	37	-	22
	Laparoscopic	-	4	52	1	35	-	21
Previous abdominal surgery	Robotic	-	-	8	9	43	-	-
	Laparoscopic	-	-	13	9	4	-	-

The effect measure for dichotomous data was odds ratios (OR) with 95% confidence intervals (CIs), whereas mean differences (MD) with 95% CI were used for continuous data. One study presented the outcome as median with the presence of the data range; therefore, we calculated means and standard deviations using Hozo’s equation [20].

The data analysis was carried out using the Review Manager software version 5.4 and all statistical results with p-values ≤ 0.05 were considered statistically significant.

Results

A total number of 2068 articles were identified in the databases. After the title screening, 154 articles were chosen, and after removal of duplicates, 138 articles remained. Ten papers comparing robotic and laparoscopic methods of APR were identified. However, only seven studies were included following full-text screening. It was found that four studies significantly overlapped, but the most recent one has been included in this review. All the included articles were published between 2015 and 2024.

The quality assessment for six observational studies was done using the Newcastle-Ottawa Scale, which showed they are of high quality [21]. The included randomized controlled trial assessed using the Revised Cochrane risk-of-bias assessment for randomized trials indicated a low probability of bias [22].

The seven studies included 10,331 patients: 8,895 in laparoscopic APR and 1,499 in robotic APR [13-19]. All studies reported baseline data on age and sex; however, four studies reported BMI [15-19] and five included ASA score [14-17,19]. All studies included comorbidities except two studies, with diabetes mellitus, hypertension, and respiratory disease being the most commonly reported comorbidities [14-15]. Three studies mentioned previous abdominal surgeries [15-17] while five studies reported the use of neoadjuvant chemoradiotherapy [14-17,19].

In four studies, TNM staging was recorded prior to surgery [14,16-18], however, it has been reported in all studies after surgery except in one study [13]. One study particularly included only rectal adenocarcinoma as part of the inclusion criteria [14], whereas three studies described the tumors’ distance from the anal margin [16,17,19].

Oncological Outcomes

Tumor size: Only three studies reported the postoperative tumor size [16,17,19]. However, one of them did not mention the standard deviation which is why it was not estimated [16], and the results of the other two studies' data analysis showed no statistically significant difference between the robotic and the laparoscopic group with P = 0.98. Postoperative TNM staging showed no significant difference between the patients who had laparoscopic and robotic surgery. Table 3 shows the TNM staging for all studies.

**Table 3:** TNM staging

Staging	No of studies	Robotic	Laparoscopic	Odds ratio	95%CI	P-value
T1	4	14/264	20/267	0.57	(0.29,1.12)	0.10
T2	5	92/286	105/288	0.85	(0.60,1.20)	0.35
T3	5	152/286	145/288	1.14	(0.82, 1.58)	0.45
T4	4	13/275	8/277	1.36	(0.55,3.35)	0.51
N0	5	180/286	188/288	0.89	(0.63,1.24)	0.49
N1	5	61/286	63/288	1.00	(0.67,1.49)	1
N2	5	38/286	32/286	1.15	(0.70,1.90)	0.58
M0	2	71/79	80/83	0.34	(0.09,1.34)	0.12
M1	2	8/79	3/83	2.94	(0.75,11.57)	0.12

**Figure 2:**
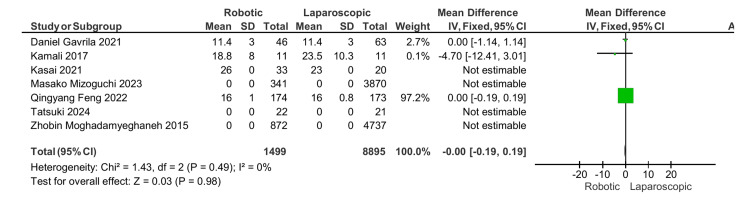
Number of harvested lymph nodes [13-19]

Number of harvested lymph nodes: In four studies, the number of harvested lymph nodes in the APR was reported [14-17]. One study was not estimated as it mentioned the MD without SD [16], and the remaining three that were included in the statistical analysis showed no significant difference with an MD of 0, 95% CI (-0.19, 0.19), and p-value of 0.98 (Figure 2).

**Figure 3:**
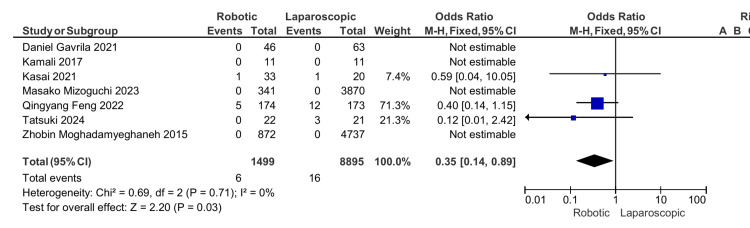
Positive resection margins [13-19]

Positive resection margin: Of all studies, only three reported the number of patients who had a positive resection margin after the operation [16,17,19]. Figure 3 shows that the positive resection margin was statistically significantly higher in the laparoscopic group with an OR of 0.35, 95% CI (0.14, 0.89), and p-value of 0.03

Postoperative Outcomes

**Figure 4:**
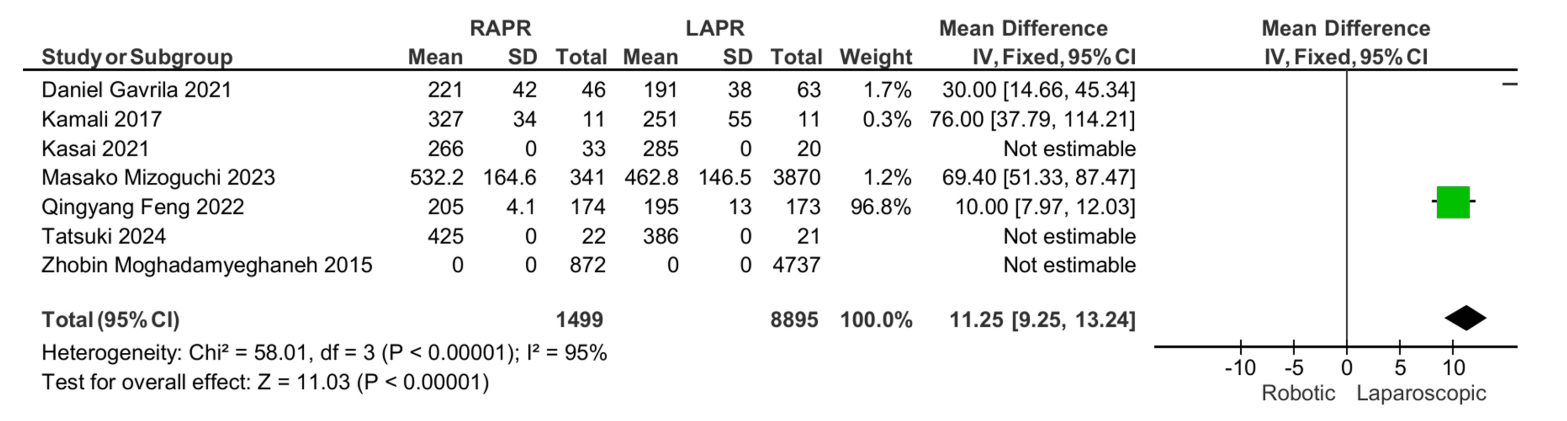
Operative time [13-19]

Operative time: A longer operation time for robotic APR compared to laparoscopic resection was reported in six studies, which was statistically significant with an MD of 11.25, 95% CI: (9.25, 13.04), and a p-value of 0.0001 (Figure 4).

**Figure 5:**
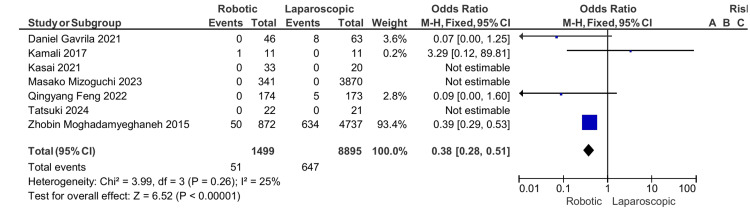
Conversion to open surgery [13-19]

Conversion rate: In six studies, conversions to open surgery were documented. When comparing robotic APR to laparoscopic APR, the conversion rate is lower with an odds ratio of 0.38, 95%CI (0. 28, 0.51), and a p-value of 0.00001, which indicates a significant difference (Figure 5).

**Figure 6:**
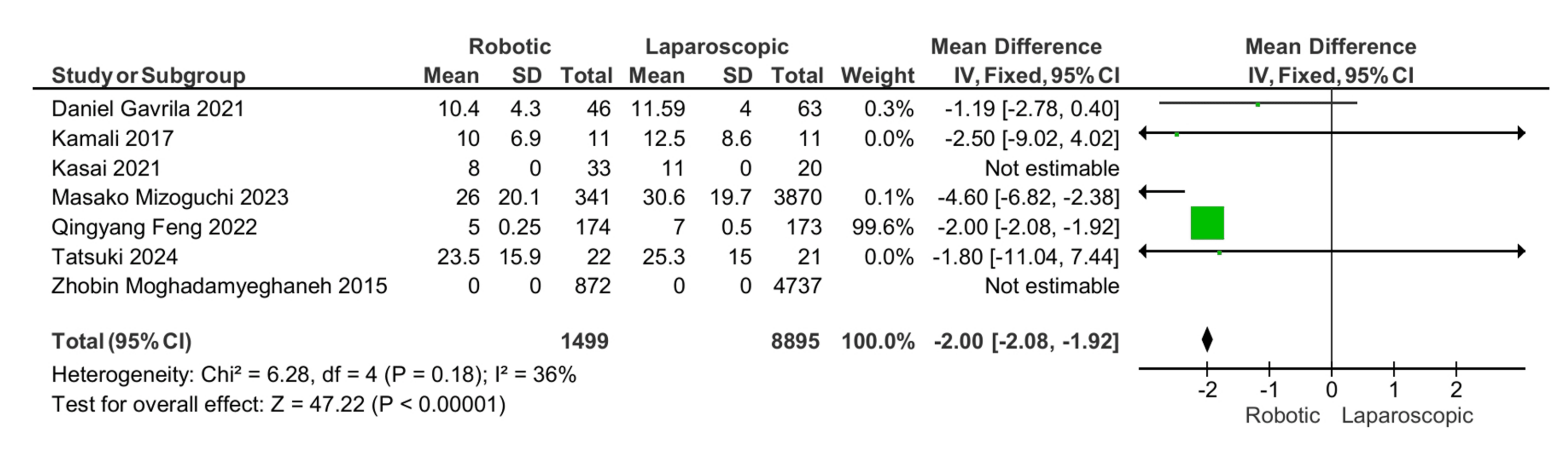
Length of hospitalization. [13-19]

Length of hospital stay: Robotic APR leads to a shorter postoperative hospital stay compared to laparoscopic APR. Five studies had an estimated MD of -2, 95% CI (-2.08, -1.92), and a p-value less than 0.00001, which is statistically significant (Figure 6).

**Figure 7:**
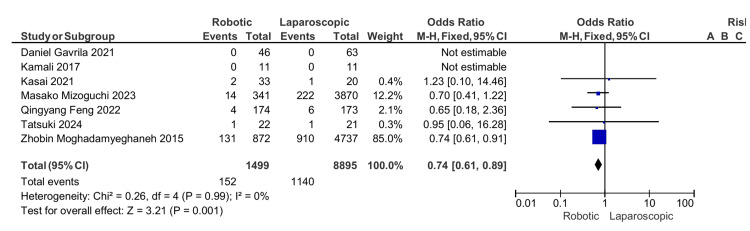
Paralytic ileus [13-19]

Paralytic ileus: It was reported in five of the seven studies, and we found that there is a higher incidence of postoperative ileus in the laparoscopic group compared to the robotic group, which is statistically significant with an odds ratio of 0.74, 95% CI (0.61,0.89), and a p-value of 0.001 (Figure 7).

**Figure 8:**
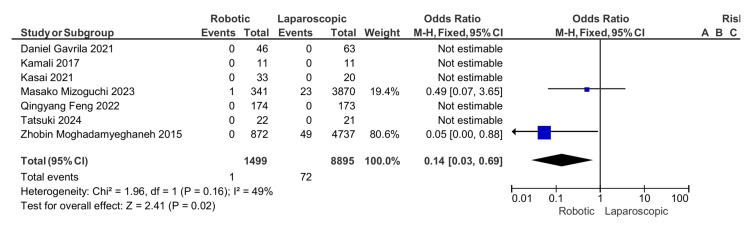
Thromboembolic complications [13-19]

Thromboembolic complications: There is a significant difference in the incidence of venous thromboembolic complications, which is less common in the robotic APR with an odds ratio of 0.14, 95% CI (0.03, 0.69), and a p-value of 0.02 (Figure 8).

Surgical site infection: Six studies indicated a difference in surgical site infections between robotic and laparoscopic APR, although it was not statistically significant [13,15-19] with an odds ratio of 1.05, 95% CI (0.79, 1.39), and p-value of 0.75.

Cardiac complication: The incidence of cardiac complications was higher in the laparoscopic group compared to the robotic group. This difference was not statistically significant, with an odds ratio of 1.6, 95%CI: (0. 86, 1.85), and a p-value of 0.24.

Pulmonary complications: There was no significant difference between the incidence of respiratory complication between the robotic and laparoscopic groups with an odds ratio of 0.97, 95% CI of (0.71, 1.34), and a p-value of 0.87.

Urinary dysfunction: Postoperative urinary dysfunction was reported in three studies [14,15,19]. We found no significant difference between robotic and laparoscopic APR, with mean differences of CI 1.01 (0.40, 2.60) and a p-value of 0.98. Table 4 summarizes the postoperative complications. 

**Table 4:** Summary of postoperative complications

Complications	Robotic n(%)	Laparoscopic n(%)	Odds ratio	95%CI	p-value
Conversion rate	51/1158 (4.4)	647/5025 (12.8)	0.38	(0.28, 0.51)	0.00001
Paralytic ileus	152/1442 (10.5)	1140/8821 (12.9)	0.74	(0.61, 0.91)	0.001
Surgical site infection	64/1488 (4.3)	323/8884 (3.6)	1.05	(0.79,1.39)	0.75
Thromboembolic events	1/1235 (0.08)	72/8628 (0.8)	0.14	(0.03, 0.69)	0.02
Cardiac complications	35/1420 (2.4)	129/8812 (1.4)	1.6	(0.86,1.85)	0.24
Pulmonary complications	47/1420 (3.3)	267/8812 (3)	0.97	(0.71,1.34)	0.87
Urinary dysfunction	9/79 (11.3)	11/95 (11.5)	1.01	(0.40,2.60)	0.98
Mortality	0/1466 (0)	54/8875 (0.6)	0.15	(0.03, 0.84)	0.03

**Figure 9:**
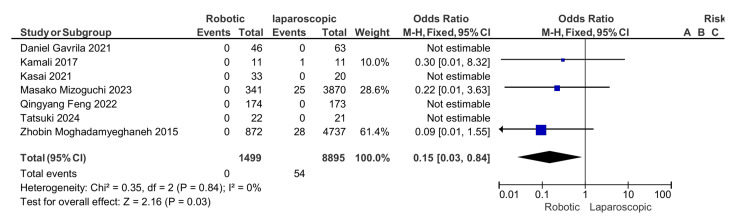
Mortality rate [13-19]

Mortality rate: Apart from one study [16], all studies revealed an increase in mortality rates in laparoscopic APR compared to robotic APR with an odds ratio of 0.15, 95% CI (0. 03, 0.84), and a p-value of 0.03 (Figure 9)

Discussion

Minimally invasive surgery has made a significant difference in colorectal surgery as it enables surgeons to perform complex resections with greater ease. Although there is a learning curve to master robotic surgery, research has shown surgeons can rapidly acquire competence [23].

Minimally invasive surgery for colorectal cancer is equally effective as open surgery in terms of achieving adequate cancer resection in regular healthcare but has better short-term results, including reduced blood loss, shorter hospital stays, a reduced mortality rate, and a lower re-admission rate [24].

This systematic review and meta-analysis was conducted to compare the oncological and short-term outcomes between robotic and laparoscopic approaches of APR, as there are multiple reviews that have explored the difference between the two approaches of rectal resection in general but no one for APR specifically [25,26]. There are a few papers comparing robotic and laparoscopic APR, and only one RCT in the databases, which is one of the limitations we have faced during this meta-analysis.

In terms of oncological outcomes, our results showed there is no significant difference between robotic and laparoscopic APR in terms of total number of harvested lymph nodes; however, the robotic group has a significantly lower rate of positive resection margin compared to the laparoscopic group. Notably, there was no difference in the TNM staging and tumor size between both study samples. In contrast, most of the studies found that there is no difference in the oncological outcomes between robotic and laparoscopic rectal surgery [16,17,19].

A single center propensity score-matched cohort study comparing laparoscopic and robotic surgery for CRC conducted in Ireland showed that there is no significant difference in the recurrence rate between the two groups in an 11-month follow-up for the laparoscopic group and eight-month follow-up for the robotic group. However, this is not specific for APR and includes all types of CRC resection [27].

We do not have any result in the recurrence rate as the studies included in this meta-analysis did not describe it, and there was no long-term follow-up for the patients. A landmark trial called ROLARR [28], which used conversion rate as the key endpoint to determine the feasibility of performing total mesorectal excision (TME), was unable to demonstrate the advantage of the robotic approach. This trial was challenged since the surgeons who performed the robotic surgery had variable levels of skills [29,30].

Other studies showed that laparoscopic surgery has a greater conversion rate compared to robotic, which is statistically significant and could be due to factors such as local tumor invasion, previous abdominal surgery, and pneumo-peritoneum intolerance. The lower rate of conversion in the robotic group could be attributed to the robotic system advantages, which offer superior three-dimensional view, better depth of perception, easier suturing, and meticulous tissue dissection in the pelvis in comparison to conventional laparoscopic surgery, and that could contribute to the platform's efficiency in lower-rectum surgery [13,15,31].

Longer operation times were seen in robotic APR procedures despite being performed by skilled surgeons in high-quality healthcare facilities, and this is consistent with most of the studies comparing both approaches of surgical resection [8,18]. After excluding the robot's docking time, some studies found no significant difference in mean operative time between groups [15]. However, multiple studies mentioned that longer operative times could be attributed to the surgical team's experience and the learning curve, which can vary greatly [32-34].

The rate of surgical site infection is reduced in robotic APR, although it is not statistically significant. Robotic APR was also found to be less likely to cause complications such as significant blood loss, paralytic ileus, urinary tract infections, and thromboembolic events, potentially leading to a shorter hospital stay. It is important to note that that shorter hospital stays, low re-admission rate, reduced complications, and conversion rate decrease treatment costs [35]. Moreover, less urinary dysfunction occurred with robotic surgery because of easier dissection of the levator ani muscle, better access to the ischiorectal fossa, steady camera, and precise operating techniques [16].

Robotic methods have some advantages over laparoscopic surgery because they enable three-dimensional vision, enhance depth awareness, have articulating wrists, eliminate the surgeon's tremor, and allow for more accurate and sophisticated movements. As a result, it enhances proficiency, makes difficult procedures easier to perform, makes tight spaces (such as the deep pelvis) easier to access, and shortens the learning curve. It is especially helpful for identifying and manipulating the surrounding organs, vasculature, and nerves during pelvic surgery. Despite this, other issues remain unresolved, including docking and surgical time, as well as the lack of tactile sense [36].

Limitations

We have faced several limitations while conducting this meta-analysis. Firstly, there are limited studies comparing robotic and laparoscopic APR, and the outcomes are not reported equally in all included studies. Secondly, some of these studies were conducted more than a long time ago with a smaller number of robotic operations compared to laparoscopic ones. Lastly, as we did not find studies comparing the long-term outcomes, we could not assess the difference in recurrence rate between the two groups.

## Conclusions

Our findings indicate that robotic APR has a better oncological and short-term outcome than laparoscopic APR. As the high incidence of rectal cancer necessitates the application of the most cost-effective and efficient strategy of management, patients with rectal cancer undergoing APR would benefit from using the robotic approach. However, more research and prospective randomized controlled trials are needed to determine the efficiency, cost-effectiveness, recurrence, and survival rate of patients undergoing robotic APR.
